# Programmable NIR Responsive Nanocomposite Enables Noninvasive Intratympanic Delivery of Dexamethasone to Reverse Cisplatin Induced Hearing Loss

**DOI:** 10.1002/advs.202407067

**Published:** 2024-11-21

**Authors:** Rawand A. Mustafa, Jiali Wang, Mengzhao Xun, Jessica M. Rosenholm, Wuqing Wang, Yilai Shu, Hongbo Zhang

**Affiliations:** ^1^ ENT Institute and Department of Otorhinolaryngology Eye & ENT Hospital Fudan University Shanghai 200031 P. R. China; ^2^ Pharmaceutical Sciences Laboratory Faculty of Science and Engineering Åbo Akademi University Turku 20520 Finland; ^3^ Turku Bioscience Centre University of Turku and Åbo Akademi University Turku 20520 Finland; ^4^ NHC Key Laboratory of Hearing Medicine Research Shanghai 200031 P. R. China; ^5^ State Key Laboratory of Medical Neurobiology and MOE Frontiers Center for Brain Science Fudan University Shanghai 200031 P. R. China; ^6^ Institutes of Biomedical Sciences Fudan University Shanghai 200032 P. R. China

**Keywords:** gold nanorod, hearing loss, intratympanic administration, local drug delivery, mesoporous silica, NIR‐responsive

## Abstract

Local intratympanic drug delivery to the inner ear possesses significant otological clinical promise as cisplatin‐induced hearing loss (CIHL) therapy, inducing significantly less side effects than systemic drug delivery. However, the multiple detoured barriers, round window membrane (RWM) and poorly controlled drug release hinder successful non‐invasive drug delivery through intratympanic administration (IT). Here, a novel near‐infrared (NIR) responsive nanocomposite functionalized with saponin, denoted gold nanorod@dexamethasone‐mesoporous silica‐saponin (AuNR@DEX‐MS‐saponin, NPs/DEX), is developed to enhance RWM permeation and to control the drug release spatiotemporally. First, the physiochemical properties and release profile of the synthesized nanocomposites are assessed, after which the biocompatibility of the nanocomposites and oto‐protection against CIHL are shown in vitro and in vivo. The findings demonstrated that DEX is delivered to the inner ear with high efficiency through IT, due to the permeation enhancement effect of the nanocomposite. Moreover, the nanocomposite with low dose of DEX is highly effective in recovering CIHL, attenuating hair cell loss, and alleviating synaptic ribbon damage. These findings provide insight into NIR‐responsive local delivery for inner ear illnesses.

## Graphical abstract

In this study, saponin‐coated silica@gold nanocarriers responsive to NIR radiation is developed to enhance intratympanic drug delivery for cisplatin‐induced hearing loss (CIHL) treatment. The biocompatibility and oto‐protective properties are validated both in vitro and in vivo. Additionally, the fabricated nanocomposite is improved round window membrane permeability for anti‐inflammatory drugs into the inner ear, enabling hearing recovering with low‐dose drug administration upon NIR radiation.

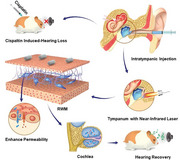

## 1 Introduction

Hearing loss is currently the most common prevalent sensory disorder globally.^[^ ^1^ ^]^ Cisplatin‐induced hearing loss (CIHL) is often observed among 40–80% tumor patients during cisplatin (Cis) treatment.^[^ ^2^ ^]^ Cis can retain at the cochlea for several months to several years, causing bilateral, progressive hearing loss and permanent damage to the hair cells, spiral ganglion, and vascular stria.^[^ ^2^, ^3^ ^]^ Previous research has demonstrated that the corticosteroid dexamethasone (DEX) exerts a protective effect against Cis‐induced ototoxicity in vitro.^[^ ^4^ ^]^ However, the drug has not shown impressive therapeutic outcome in clinical practice, due to the multiple biological barriers that prevent this drug from maintaining concentration within the therapeutical window in the inner ear. Thus, there is a significant unmet clinical need for developing drug‐delivery tools for inner ear ailments.^[^ ^5^ ^]^

Intratympanic administration (IT) of DEX, one of the most prevalent ways for drug delivery locally to the inner ear, has shown significant otological clinical promise for CIHL therapy due to increased local concentration of the drugs and reduced adverse effects compared to systemic drug delivery.^[^ ^5^, ^6^ ^]^ Nonetheless, the round window membrane (RWM) has poor permeability and the quick evacuation through the Eustachian tube result in inadequate drug concentrations, consequently reducing their effectiveness.^[^ ^7^ ^]^ Numerous strategies, including microneedles,^[^ ^8^ ^]^ chemical penetration enhancers,^[^ ^7b^ ^]^ hydrogels,^[^ ^9^ ^]^ nanoparticles,^[^ ^10^ ^]^ microparticles,^[^ ^11^ ^]^ and nanoparticle‐loaded hydrogel,^[^ ^12^ ^]^ have been introduced to overcome such obstacles. Nonetheless, these systems have shown variable abilities to penetrate the middle‐inner ear barriers, still appearing to be insufficient to achieve efficient clinical outcomes. Every approach remains vulnerable to anatomical restrictions, as drug flux is required to diffuse passively over the RWM barrier.^[^ ^13^ ^]^ In addition, the periodic utilization of Cis and the uncontrollable release of the drug have led to a further decline in its therapeutic effect.^[^ ^2b^ ^]^ Thus, a remote‐control release vehicle that can enhance drug concentration at the target site would be necessary for effective IT therapy.

Previous studies have used intrinsic stimuli (pH, ROS, and temperature) and external stimuli (magnetic field and ultrasound) to alter the release rate of drugs, increase the permeability of RWM, and enhance the efficacy of drug in treating hearing impairment.^[^ ^14^ ^]^ Considering the limited availability and effectiveness of intrinsic stimuli, the more controllable external stimuli can spatiotemporally change the permeability of RWM and thus improve drug delivery efficacy. Among these, the release of drug by biocompatible near‐infrared laser (NIR), which offers superior tissue transmission, minimal side effects, and provides an excellent opportunity to deliver and activate a therapeutic agent at the precise spatiotemporal location.^[^ ^15^ ^]^ Studies have reported that NIR stimulation can affect the activity of cytochrome C oxidase and thus inhibit inflammation and apoptosis, and the pretreatment of near‐infrared stimulation can reduce the damage of hair cells and nerves caused by noise and cochlear implantation.^[^ ^16^ ^]^ NIR can be used as an on/off switch button to control the release of dexamethasone spatiotemporally, which is expected to improve the efficacy of dexamethasone for CIHL therapy.

**Scheme 1:**
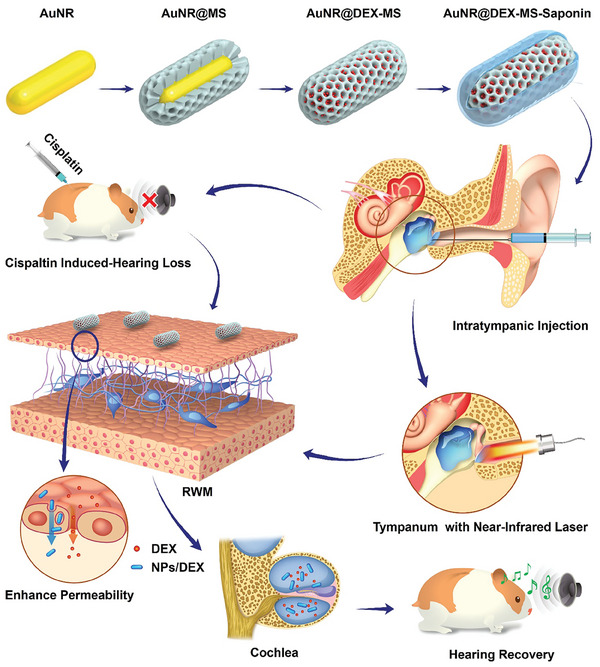
Schematic depiction of the developed nanocomposite AuNR@DEX‐MS‐Saponin (NPs/DEX) and the IT delivery of nanocomposite for the CIHL therapy. Nanocarrier based on NIR laser light can enhance RWM permeability of DEX into the inner ear and trigger the drug release.

In this study, we developed an intratympanic delivery vehicle with NIR responsive remote control and permeability enhancer function, as illustrated in **Scheme** **1**. Gold nanorods (AuNRs) enclosed in a mesoporous silica (MS) layer were utilized as a multifunctional delivery system with NIR induced photothermal conversion. The MS serves as a reservoir for corticosteroids, whereas the AuNR core has the capability to undergo photothermal conversion to increase drug release and promote RWM permeability. Saponin, an amphiphilic molecule, performs as a permeability enhancer by removing membrane cholesterol and phospholipids, thereby weakening the lipid bilayer‐structured barrier.^[^ ^17^ ^]^ There have been previous reports showing that IT of saponins can enhance the drug penetration through RWM, thus increase the concentration of DEX in the perilymph.^[^ ^18^ ^]^ Consequently, saponin was employed to coat DEX‐loaded AuNR@MS core‐shell nanoparticle (AuNR@DEX‐MS‐saponin, NPs/DEX). When targeted NIR radiation is applied externally to the inner ear, the heat generated by AuNR core enhances the drug penetration through the RWM and accelerates the DEX release from the MS shell which acts as a drug reservoir. In addition, saponin could further boost RWM permeability and control the drug release by acting as a coating on top of the nanocomposite surface. As a result, the developed nanocomposite has the potential to enhance RWM permeability via synergistic combination of NIR radiation and controlled drug release to effectively treat CIHL.

## 2 Results and Discussion

To enhance the therapeutic effect of DEX through IT, we exploited an NIR‐based nanocomposite with saponin properties as a permeability booster (NPs/DEX) to promote the RWM permeability of DEX through synergistic action, as shown in Scheme 1. Herein, saponin as permeability enhancer was employed to modify the surface of AuNR@DEX‐MS. Through synergistic action of the heat generated by AuNR core and saponin property, the permeability of the RWM could be concurrently increased. In particular, the AuNR core‐generated heat accelerates DEX release via NIR from the MS shell as shown in Scheme 1. In this way, there will be a significant accumulation of DEX within the inner ear, thereby providing an adequate amount for the targeted cells.

### 2.1 Characteristics of NPs/DEX

**Figure 1:**
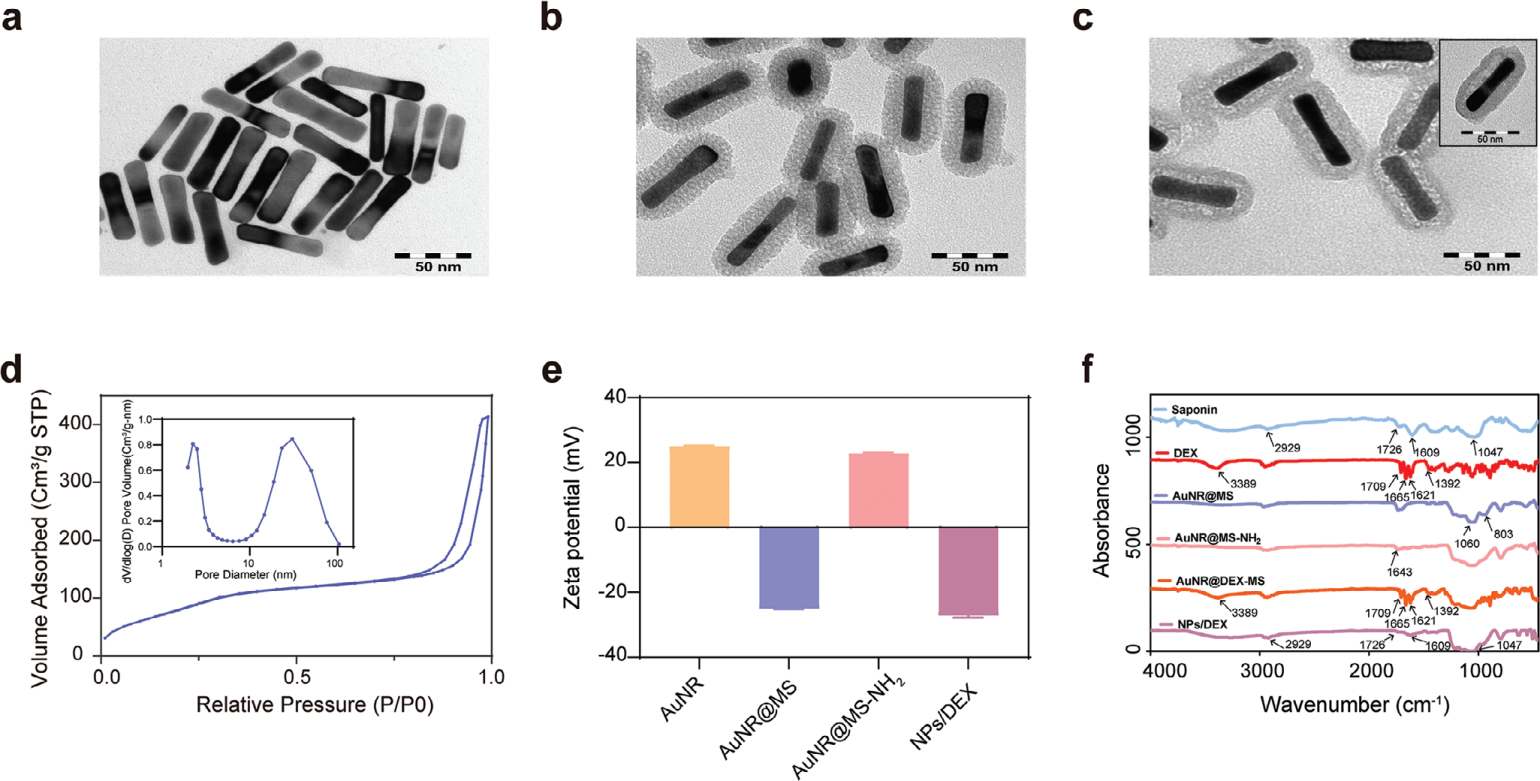
Characterization of the nanoparticle. a, b, c, TEM images (Scale bar = 50 nm) of a) AuNR, b) AuNR@MS, and c) NPs/DEX. d) the relevant N_2_ adsorption–desorption isotherms reveal the distribution of pores in AuNR@MS. e) Zeta potential of AuNR, AuNR@MS, AuNR@MS‐NH_2_, and NPs/DEX. f) FTIR spectra of Saponin, DEX, AuNR@MS, AuNR@MS‐NH_2_, AuNR@DEX‐MS, and NPs/DEX.

As it is shown in **Figure** **1**a, demonstrates that AuNRs have a homogeneous and well‐dispersed rod‐like structure. The average breadth is 12.22 ± 0.94 nm, average length is 64.45 ± 1.94 nm, and the aspect ratio is around 5. The AuNRs were enclosed by a silica shell with a mesoporous structure and a thickness of 12.19 ± 0.45 nm for the loading of DEX and further coating with saponin (Figure 1b,c). Since, the MS layer has the potential to improve the AuNR colloidal stability, thereby increasing the NIR light's transmission in biological tissue.^[^ ^19^ ^]^ In addition, saponin was utilized as a permeability enhancer to encapsulate the synthesized AuNR@MS and form the hybrid nanocomposite (NPs/DEX), as shown in Scheme 1. A thin polymer layer is clearly visible in Figure 1c, and the silica pores disappear from the surface. The nitrogen adsorption–desorption isotherms and pore size distribution curves of AuNR@MS were determined using Brunauer–Emmett–Teller analysis. The pore size distribution was mostly 2.3 nm, and the specific surface area reached 308 m^2^ g^−1^ (Figure 1d). These findings revealed that the nanoparticles possessed adequate volume and surface area for effective drug loading. The hydrodynamic size of the synthesized AuNR@MS, AuNR@MS‐NH_2_ and NPs/DEX were 134.70 ± 1.22, 142.10 ± 0.56, 162.57 ± 1.20 respectively, and the PDI were respectively 0.02 ± 0.02, 0.04 ± 0.01, and 0.12 ± 0.20, which is confirmed as a homogenous distribution, as it shown by the dynamic light scattering (DLS) (Figure S1a,b, Supporting Information). Additionally, as shown in (Figure S2a–c, Supporting Information), the narrow size distribution peak confirms the monodispersity of the synthesized AuNR@MS, AuNR@MS‐NH_2,_ and NPs/DEX. Furthermore, ζ‐potential was assessed to demonstrate the synthesis result of the nanocomposite NPs/DEX. In Figure 1e, the AuNR core exhibited a positive ζ‐potential value, attributed to the presence of CTAB on its surface (25.07 ± 0.15 mV), while a significantly negative ζ‐potential was measured when the AuNR core was enclosed in a silica shell AuNR@MS (‐25.03 ± 0.15 mV) due to the synthesis of copious Si─OH.^[^ ^15^ ^]^ In addition, the ζ‐potential of AuNR@MS was substantially changed to the positive after modification with ─NH_2_ (22.80 ± 0.26 mV) and became a very negative ζ‐potential following coating with the polymer layer (−27.20 ± 0.62mV) because the sugar residues have carboxyl groups,^[^ ^20^ ^]^ resulting in the development of the nanocomposite NPs/DEX. Furthermore, the AuNR@MS sample shows band at 1060 and 803 cm^−1^ in FTIR, which corresponds to the bending vibration of Si─O and stretching vibration of Si─O─Si, respectively. This proves that the MS coating of the AuNR is successful (Figure 1f),.^[^ ^21^ ^]^ Additionally, we detected a vibrational band, which is associated with amine group N─H at 1643 cm^−1^, that proves the successful NH_2_ surface functionalization. Further, the spectra of DEX exhibit distinctive bands at 1395 and 3389 cm^−1^, attributed to C─F and O─H vibrational stretching, respectively. The bands at 1709, 1665, and 1621 cm^−1^ represents C═O conjugated to C═C and was also observed in the AuNR@DEX‐MS sample.^[^ ^22^ ^]^ For the saponin spectra, the band at 1609 cm^−1^ is associated with the stretching vibration of the C═C bond, while the band at 1065 cm^−1^ corresponds to the symmetric stretching of the lipids CO─O─C (ester). Additionally, the band at 1726 cm^−1^ is attributed to the stretching band of C═O (carbonyl) groups.^[^ ^23^ ^]^ Moreover, the band at 2929 cm^−1^ corresponds to the antisymmetric stretching vibration of the saturated ─CH_2_. The FTIR data revealed that saponin was effectively coated onto the AuNR@DEX‐MS to create NPs/DEX.

### 2.2 Assessment of the Photothermal Properties of NPs/DEX

**Figure 2:**
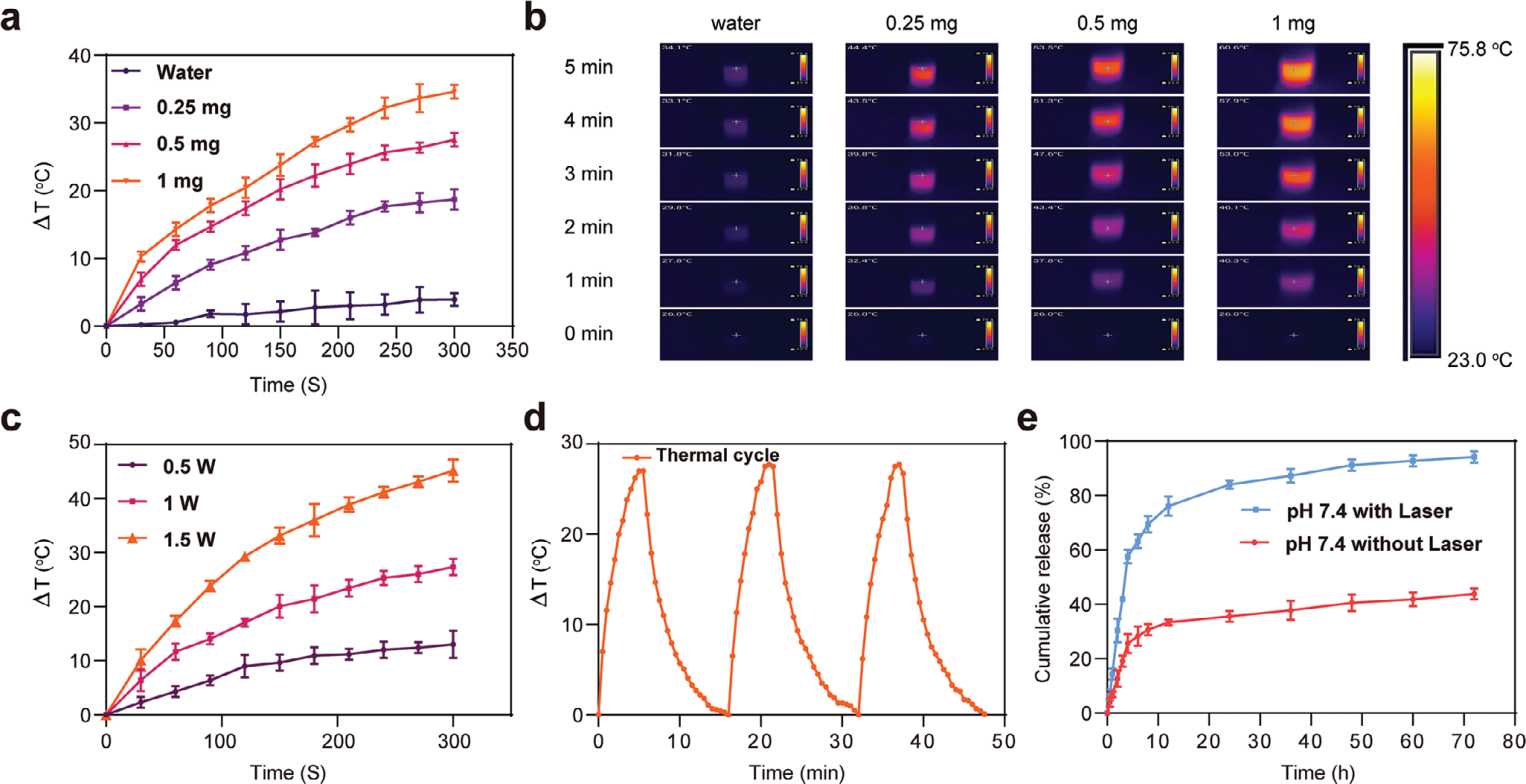
Characterization of photothermal effects and cumulative DEX release profiles of synthesized NPs/DEX. a) Concentration‐dependent photothermal heating curves of DI water and synthesized NPs/DEX at (0.25, 0.5, and 1 mg mL^−1^), under 1.0 W cm^−2^, 980 nm laser irradiation (1.0 W cm^−2^, 0–300 s, 26 °C); water served as a control. b) Infrared thermal pictures of nanocomposite and DI water at different concentrations exposed to a (980 nm, 1.0 W cm^−2^, 300 s). c) Photothermal heating curves of a synthesized nanocomposite 0.5 mg mL^−1^ were exposed to laser of different densities (0.5, 1, and 1.5 W cm^−2^) for a duration of 0–300 s. d) The effect of three cycles of NIR on/off irradiation (1.0 W cm^−2^, 980 nm) on the temperature increase of the synthesized nanocomposite (0.5 mg mL^−1^). e) Synthesized NPs/DEX release profile in response to laser irradiation.

The photothermal characteristics of produced nanoparticles were evaluated under 980 nm of light source radiation. The quantitative temperature variation of NPs/DEX in aqueous solutions in response to concentration, laser exposure period, and power density was investigated. Several concentrations (0.25, 0.5, and 1 mg mL^−1^) of NPs/DEX were exposed to irradiation by an NIR laser (980 nm, 1 W cm^−2^, 300 s) as shown in **Figure** **2**a,b. The temperature changes negligibly in pure water regardless of the duration of irradiation. While the temperature increases dramatically as NPs/DEX concentration and irradiation period increase and reached to 60.6 °C at 1 mg mL^−1^, 300 s (Figure 2a). Moreover, the photothermal images indicate that the nanoparticles convert NIR energy to heat more efficiently than the control (water) following an equivalent laser exposure length (Figure 2b).^[^ ^24^ ^]^ To investigate the impact of laser power density on temperature rise, 0.5 mg mL^−1^ of NPs/DEX was subjected to NIR lasers for 300 s at varying power density (0.5, 1.0, and 1.5 W cm^−2^) (Figure 2c). The rate of temperature rise and ultimate temperature were directly proportional to the laser power intensity.^[^ ^25^ ^]^ NPs/DEX indicated a distinct photothermal response dependent on laser power, with temperature rising to 28 and 46 °C at (1.0 and 1.5 W cm^−2^), respectively. The results revealed that the temperature increase was due to the photothermal impact of AuNR.

Besides the photothermal conversion efficiency, the photostability of the fabricated nanocomposite NPs/DEX were examined as a crucial property for an effective carrier to release drug using three heating‐cooling cycles (Figure 2d). The results indicated that the temperature rise was efficiently sustained during the three cycles examined. After each cycle of laser irradiation, the temperature of nanocomposite increased at an accelerate rate and reached a plateau, indicating that NPs/DEX is capable of a very stable photothermal conversion. The photothermal conversion efficiency of NPs/DEX was assessed to be 26.3% based on the heating–cooling curves in (Figure 2d; Figure S3, Supporting Information). The (*η*) was determined using Equations (1) and (2) in the following manner:

(1) $$\eta =hs\Delta T_{\max}-Q/I\left(1-10^{-A980}\right)$$

In this equation, ΔTmax signifies the maximum change in temperature, *Q* refers to the temperature increase of the solvent, and *I* represents the power of laser. A^980^ denotes the absorbance of NPs/DEX at 980 nm. hs, on the other hand, can be calculated using the subsequent formula:

(2) hs=mc/τ

The letters *m* and *c* represent the mass of the solution and specific heat capacity, respectively. The fitted slope from *τ* to ‐ln ΔT/ΔT max is denoted by τ.^[^ ^26^ ^]^ Moreover, According to DEX loading content assessment, an appropriate ratio of nanoparticles (1:2; AuNR@MS:DEX) was used, resulting in loading efficiency of up to 30% after coating, as measured by UV–vis spectroscopy. The DEX release investigation was conducted in PBS (pH 7.4, 37 °C). As depicted in (Figure 2e), the DEX release from the nanocomposite NPs/DEX, following irradiation and heating up with a 980 nm NIR laser, revealed an initial 76.96% of total DEX burst release during the first 12 h. It was then steadily released until it approached a maximum level of 94.17%. In the meantime, in the absence of laser, only 33.43% of DEX was released in 12 h, and this percentage increased to merely 43% in the tested period. The result revealed that NPs/DEX is capable of controlled and enhanced DEX release induced by NIR.

### 2.3 Cytotoxicity and Otoprotective Properties of NPs/DEX

**Figure 3:**
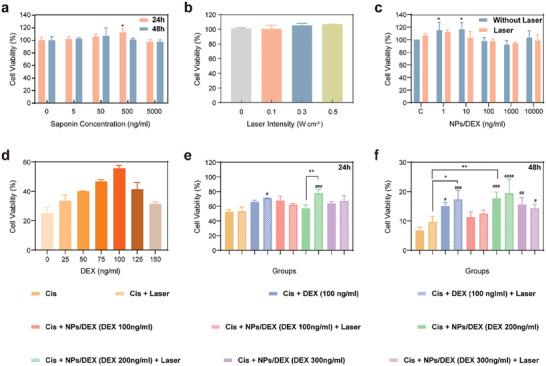
In vitro cell viability of HEI‐OC1 cells treated with saponin, DEX, synthesized NPs/DEX at varying concentrations and different laser density. a) HEI‐OC1 cell viability in saponin at different concentrations. b) HEI‐OC1 cell viability in different laser intensity. c) HEI‐OC1 cell viability NPs/DEX with and without laser at different concentrations. d) HEI‐OC1 cell viability at different DEX concentrations. e) and f), The viability of HEI‐OC1 cells cultivated with DEX and NPs at various concentrations against 30 µm Cis in 24 h (e) and 48 h (f). (*n* = 3, ^####^: *p* < 0.0001, ^###^: *p* < 0.001, ^**^, ^##^: *p* < 0.01, and ^*^, ^#^: *p* < 0.05).

To improve the therapeutic effect of the DEX, the NIR‐based nanocomposite‐the AuNR core coated with MS and saponin, loaded with DEX, were exploited for boosting the permeability of the RWM. In the **Figure** **3**, The biocompatibility of the saponin, DEX, NPs, and laser were evaluated independently by the cell viability of HEI‐OC1, and the cell viability in each group exhibited concentration and laser intensity‐dependent. As shown in Figure 3a, the saponin at certain concentrations (5, 50, 500, and 5000 ng mL^−1^) didn't show significant cytotoxicity at 24 h. The cell viability of the HEI‐OC1 after 980 nm laser for certain duration and different intensity were assessed, and no cytotoxicity are shown in Figure 3b. Thus, NIR laser shows good biocompatibility to HEI‐OC1. Furthermore, the cell viability showed no significant difference after NPs/DEX (1, 10, 100, 1000, and 10 000 ng mL^−1^) co‐incubation with/without NIR laser (0.3 W cm^−2^, 10 s). To evaluate the otoprotective efficacy of the NPs/DEX against Cis – induced HEI‐OC1 cell damage, we use 30 µM Cis as an appropriate concentration to induce HEI‐OC1 cell damage. DEX demonstrates otoprotective effect against Cis – induced HEI‐OC1 cell damage, and 100 ng mL^−1^ was shown as the optimal concentration (Figure 3d). Subsequently, as shown in Figure 3e,f, it revealed that NPs/DEX protected HEI‐OC1 cells from Cis‐induced damage. The cells were pretreated using DEX alone or NPs/DEX loaded with various concentrations of DEX for 5 h with /without laser and then after 1 h damaged with Cis for an additional 24 or 48h. We discovered that pretreatment with NPs/DEX at a commensurate degree irrespective of DEX concentration could inhibit Cis‐induced cytotoxicity with/without laser. Notably, the DEX with NIR laser showed more protective effect than that the DEX alone. Moreover, HEI‐OC1 cells exposed to the NPs/DEX nanocomposite contained 200 ng mL^−1^ DEX had relatively higher cell viability as compared to the DEX treated group with NIR laser for 24 and 48 h. However, in the group that is not treated with NIR, HEI‐OC1 cell viability was decreased but not less than 50% as compared to NPs/DEX or DEX groups. Consequently, the results of the cell investigation suggest that the synthesized NPs/DEX could be used to further evaluate its potential in hearing loss treatment that is induced by Cis.

### 2.4 Hearing Loss Evaluation of NPs/DEX

**Figure 4:**
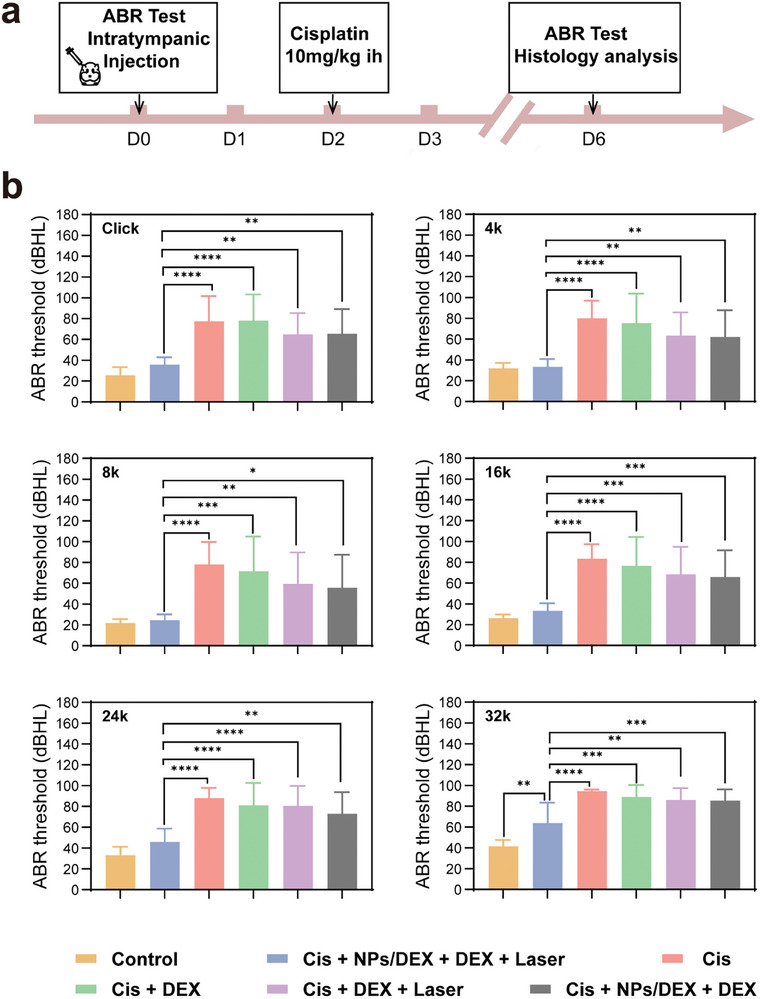
The ABR threshold of guinea pigs, both before and after IT. a) Schematic of the ABR test schedule for guinea pigs. b) ABR threshold values on day 4 following Cis excessive exposure at various frequencies in the control, Cis + NPs/DEX+DEX + Laser, Cis, Cis + DEX, Cis +DEX + Laser, and Cis + NPs/DEX+DEX groups, 980 nm, 0.3 W cm^−2^ for 10 s was used for laser group (n _control_ = 8, n _Cis_ = 10, n _Cis + DEX_ = 10, n _Cis +DEX + Laser_ = 10, n _Cis + NPs/DEX+DEX_ = 12, n _NPs/DEX+DEX + Laser_ = 12; ^****^ *p* < 0.0001, ^***^ *p* < 0.001, ^**^ *p* < 0.01, and ^*^ *p* < 0.05).

The outer hair cells (OHCs) and the synaptic ribbons of inner hair cell (IHC) region are easily vulnerable with Cis administration.^[^ ^27^ ^]^ As shown in the timeline (**Figure** **4**a), auditory brainstem response (ABR) thresholds, OHCs loss rate, and the ribbons number per IHC region were evaluated to demonstrate the CIHL in guinea pigs before and after inner ear therapy (Figure 4a). The guinea pigs were divided into the following groups: Cis, Cis + DEX, Cis + NPs/DEX, Cis + NPs/DEX + DEX, Cis + NPs + DEX, and Cis + DEX + Laser, Cis + NPs/DEX + Laser, Cis + NPs/DEX + DEX + Laser, control (Figure S4c). Negative control group received sham IT with no exposure to Cis damage. The other groups respectively received IT of saline (Cis group), DEX solution with or without laser (Cis + DEX + Laser / Cis + DEX group), NPs/DEX with DEX solution with or without laser (Cis + NPs/DEX + DEX + Laser / Cis + NPs/DEX + DEX group), NPs/DEX with DEX solution with or without laser (Cis + NPs/DEX / Cis + NPs/DEX + Laser), NPs with DEX solution (Cis + NPs + DEX) two days before Cis (10 mg Kg^−1^) hypodermic injection. The guinea pigs which only received Cis hypodermic injection had the greatest effect at all frequencies (4, 8, 16, 24, 32 kHz, and click). On day 4 after Cis injection, the ABR thresholds of the Cis group were 81.87 ± 14.37, 80.62 ± 18.60, 83.75 ± 14.33, 88.12 ± 10.32, 94.37 ± 1.76, and 81.25 ± 19.22 dB SPL at 4, 8, 16, 24, 32 kHz, and click, respectively (Figure 4b). The inner ear for the untreated guinea pigs exhibits severe hearing loss when exposed to Cis. Therefore, the inner ear treatment is essential as Cis accumulation can induce such urgent and severe hearing loss. In addition, the ABR of other treated groups all decline which demonstrates varying degrees of the protective effect of the DEX in different forms. Moreover, NIR seems to enhance the therapeutic impact of DEX, as the ABR thresholds of Cis + DEX + Laser group is better than Cis + DEX group (63.5 ± 22.24, 59.5 ± 30.13, 68.5 ± 26.36, 80.5 ± 19.21, 86.0 ± 11.50, and 64.9 ± 20.31 versus 75.5 ± 28.23, 71.5 ± 33.42, 76.5 ± 27.89, 81.0 ± 21.58, 89.0 ± 11.50, and 78.0 ± 25.30, at 4, 8, 16, 24, 32 kHz, and click). However, it shows no significant difference between Cis + DEX + Laser group and Cis + DEX group, and no significant protective effects relative to the Cis group at all frequencies (Figure 4b). This concludes that neither laser alone nor pure drug is capable of producing an efficient protective effect against CIHL in the guinea pig. Furthermore, the ABR thresholds of Cis + NPs/DEX + DEX + Laser group is better than Cis + NPs/DEX + DEX group (33.33 ± 7.49, 24.58 ± 5.42, 33.33 ± 7.18, 45.83 ± 12.94, 63.75 ± 19.67, and 35.83 ± 7.02 versus 62.08 ± 25.80, 55.83 ± 31.47, 65.83 ± 25.75, 72.92 ± 20.83, 85.42 ± 10.76, and 65.42 ± 23.78, at 4, 8, 16, 24, 32 kHz, and click). The ABR thresholds of guinea pigs that were administered Cis + NPs/DEX+DEX, on the other hand, revealed no significant protective effect. Notably, as illustrated in Figure 4b, the ABR of Cis + NP/DEX + DEX + Laser group has the most significant treatment effect compared to other treated groups. And the Cis + NPs/DEX, Cis + NPs/DEX + Laser, and Cis + NPs + DEX didn't show as great a treatment outcome as Cis + NPs/DEX + DEX + Laser, which suggested that NPs/DEX, DEX, and the Laser are indispensable for the therapeutic effect. Furthermore, a sufficient quantity of free DEX can permeate RWM to enhance the protective effect, resulting in the ability of NIR to increase permeability of DEX into the inner ear. Specifically, there is no statistically significant difference between control groups and the Cis + NP/DEX + DEX + Laser group in terms of hearing loss at all frequencies except for 32 kHz (*p* < 0.01). This finding confirms that the high frequency region (basal turn) is more susceptible to Cis‐induced toxicity, leading to more severe damage compared to low frequency region (apical turn). Alternatively, the observed damage may be attributable to other factors.^[^ ^28^ ^]^ This finding suggests that the NPs/DEX + DEX+ Laser group has shown a promising protective effect in CIHL guinea pigs. As it provides a synergistic effect by combining the effects of saponin as a permeate enhancer and laser to trigger on‐demand DEX release and generate heat to further enhance the permeability of DEX into the inner ear, it contributes to a therapeutically sufficient concentration of DEX into the inner ear.

**Figure 5:**
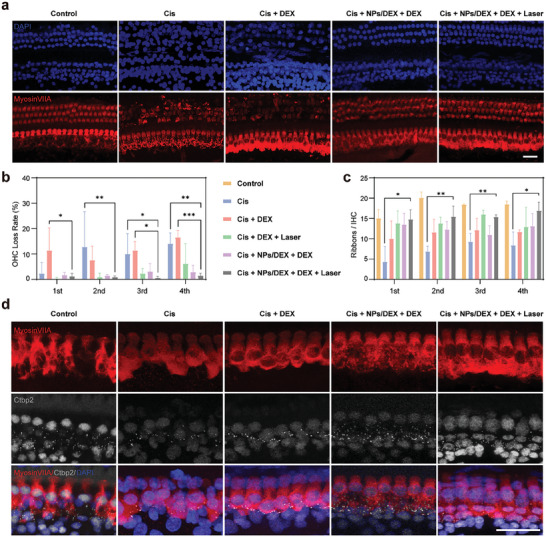
Depicts a fluorescence image of the OHCs and IHC. a) Representative immunofluorescence pictures of OHCs from each group's fourth turn (base turn), survival OHCs were visualized by fluorescent staining for their nuclei with DAPI (blue) and anti‐Myosin VIIA (red). b) Statistics regarding the rate of loss of OHCs in each group (*n* = 4; ^***^ *p* < 0.001, ^**^ *p* < 0.01, ^*^ *p* < 0.05). c) Statistical study of the number of synaptic ribbons per IHC of each turn in each group (*n* = 3; ^*^ *p* < 0.05). d) Representative immunofluorescence pictures showing CtBP2 expression in the IHC region near the proximal base turn. Immunostaining was performed for the synaptic ribbons (anti‐CtBP2, gray) and Myosin VIIA (red). Scale Bar = 25 µm.

The ototoxicity of Cis is obvious, with hearing loss occurring hours or days after the initial administration.^[^ ^29^ ^]^ Auditory is mostly dependent on the structural and functional integrity of hair cells and their accompanying spiral ganglion neurons, and abnormalities in these cells result in hearing loss or deafness.^[^ ^30^ ^]^ To further evaluate the effect of synthesized NP/DEX nanocomposite on CHIL protection, we characterized the structure and number of phalloidin‐stained OHCs with confocal microscopy. As shown in **Figure** **5**a, the number of OHCs decreased following overexposure to 10 mg Kg^−1^ of Cis on day 6 at each turn. The statistical analysis also revealed that the Cis group and the Cis + DEX subgroup had a significantly higher rate of hair cell loss when compared to the other subgroups (Figure 5b). In addition, the OHC loss in Cis + DEX + Laser reduced per turn, which is in line with the ABR. This is due to the fact that a little free DEX can permeate through RWM to enhance the protective effect of OHC. Interestingly, the OHC survival rate is increased in both the Cis + NP/DEX + DEX + Laser group and the Cis + NP/DEX + DEX group at all turn. In particular, the OHC loss rate in Cis + NP/DEX + DEX + Laser group is lower than Cis + DEX (4th, *p* < 0.001; 3rd, *p* < 0.05; 1st, *p* < 0.05) and Cis (4th, *p* < 0.01; 3rd, *p* < 0.05; 2nd, *p* < 0.01) respectively. This finding suggests that the nanoparticle approach can enhance the therapeutic effect of DEX, protect the OHC and ameliorate the OHC loss. However, the discrepancy of Cis + NP/DEX + DEX + Laser and Cis + NP/DEX + DEX group between ABR and OHC loss rates showed inconsistency. And thus, there may be other reasons lead to this inconsistency.

Immunostaining was also used to examine the presynaptic ribbon component CtBP2 and the expression of myosin VIIA in sensory hair cells following Cis hypodermic injection, to evaluate synaptic degeneration in the IHC region. We quantified synaptic ribbon loss in the IHC region by counting CtBP2 commas in the Myosin VIIA region. As exhibited in (Figure 5c,d), it is demonstrated that following Cis administration, synaptic ribbons were damaged, as there were substantially fewer synaptic ribbons per IHC of the Cis group in relation to the control group. At all turns, the number of synaptic ribbons per IHC was significantly higher in the Cis + NPs/DEX + DEX + Laser group than in the Cis group (4th, *p* < 0.001; 3rd, *p* < 0.05; 2nd, *p* < 0.001; 1st, *p* < 0.0001). Additionally, as compared to the other treated groups, the Cis + NP/DEX+DEX + Laser group exhibited a higher number (not statistically significant) of synaptic ribbons per IHC at all turns. It appears that Cis + DEX and Cis + NP/DEX + DEX do not have the potential to provide a full recovery therapy as they did not achieve the satisfied result regarding ABR threshold outcomes, despite their ability to slow the rate of OHC loss and synaptic ribbons damage caused by Cis. This may be due to other structural damage or severe damage to the sensory hair organs. Thus, reported results indicate that the produced NPs/DEX+DEX + Laser had a greater impact in mitigating hair cell loss and synaptic ribbons damage generated by Cis and enhancing ABR threshold across all frequencies except 32 kHz, this might be attributed to Cis‐induced severe injury or other organ malfunction. More specifically, synthesizing NP/DEX enhances synergistic therapy in response to laser exposure, thereby increasing drug penetration through RWM via IT.

## 3 Discussion

Ototoxicity caused by Cis is prevalent, and it always has long‐lasting effects on the inner ear. Upon receiving systemic Cis therapy, the drug enters the inner ear within minutes, and it has been proven to be vulnerable to the OHCs. Since Cis‐induced ototoxicity and hearing loss is permanent, prophylactic or early therapy is essential for effective treatment approaches.^[^ ^31^ ^]^ For inner ear illness and Cis‐induced ototoxicity, the intratympanic glucocorticoids‐based nanoparticles delivery system has already been investigated as a promising strategy for inner ear illness, including CIHL, sudden sensorineural hearing loss, and Meniere's disease. Nonetheless, their ability to penetrate RWM, which is one of the key challenges to delivering therapeutic amounts into perilymph in the inner, still has to be enhanced.^[^ ^4^, ^28^, ^32^ ^]^ Therefore, we developed NPs/DEX in this research. To our understanding, this represents the first study to demonstrate the administration of DEX via an intratympanic locally delivered NIR‐based nanocomposite with saponin characteristics for the inner ear biomedical application. By taking advantage of using AuNR‐core to generate heat upon NIR‐laser radiation to increase RWM permeability and trigger DEX release on demand in silica pore as a carrier and modifying silica coating with saponin to provide additional permeability potential. As hypothesized, the combination of these characteristics enhanced the therapeutic effect of Cis‐induced ototoxicity. The physicochemical characteristics and photothermal stability of the synthesized NPs/DEX were evaluated. The OHC based cytotoxicity evaluation revealed that the manufactured NPs/DEX regardless laser in a range of concentrations is biocompatible. In addition, NPs/DEX has a significant otoprotective effect in OHCs compared to the Cis group. In guineas treated with laser induced NPs/DEX+ DEX group, the ABR threshold was considerably lower across the frequency range compared to other Cis treated groups, and the NPs/DEX, NPs/DEX + Laser, and NPs/DEX +DEX didn't show better effectiveness than NPs/DEX + DEX + Laser, which suggested that NPs/DEX +DEX + Laser can not only improve the RWM permeability, increase the drug concentration in the inner ear, but also keep the drug sustained release. To have an immediate effect, a substantial quantity of therapeutic drug concentration must be able to travel across RWM. Applying a larger drug concentration outside of the RWM will therefore maximize RWM permeability and enhance the urgent therapeutic effect based on concentration gradients per Fick's rule. As a consequence, the DEX solution was added into the NPs formulation in our investigation. However, the rapid discharge of DEX solution through the eustachian tube presents is an additional challenge.^[^ ^12^ ^]^ Therefore, we hypothesized, when NIR laser light is utilized, it has an effect that enhances the permeability of both DEX solution and NPs, and saponin can further enhance the permeability of the RWM by offering a synergistic effect. All the optimistic results in vitro and in vivo indicated our developed nanocomposite has a potential to deliver efficient quantity of DEX into the inner ear in a controllable and sustained manner via synergism properties. Despite these strengths, there is still potential for improvement. Nanocomposite can be combined with a thermos responsive microgel, such as GelMA, to further enhance its efficacy.^[^ ^6b^ ^]^ This combination can improve the pharmacokinetic profile of dug for various forms of acquired deafness by increasing adhesion qualities and encapsulating different drug molecules that work in separate pathways. Moreover, owing to the large surface area and straightforward functionalization of the MS layer, it is possible to anchor different targeting ligands as a step toward delivering therapeutic agent to a specific target site or organ in the inner ear, or to distribute therapeutic drug molecules through all turns in the cochlea in order to provide a more effective treatment for hearing loss suffering from varying frequencies.

## 4 Conclusion

To sum up, by enclosing a silica‐coated AuNR loaded with low‐dose DEX within saponin, we were able to effectively create a synergistic nanocarrier based‐NIR delivery system for localized IT delivery to the inner ear. The AuNR core‐generated heat under NIR radiation to accelerate DEX release from the MS shell. The heat and saponin layer work synergistically to increase permeability. The synthesized nanocarrier exhibited triggered release of DEX upon NIR radiation. The synthesized nano vector exhibited biocompatibility and significant protective effects at the cellular level regardless of NIR laser against CIHL. In addition, in vivo examination revealed that the produced nanocomposite effectively restored nearly complete hearing in guinea pigs subjected to NIR radiation across all frequencies. Moreover, the ability of synthesized NPs/DEX in protecting OHCs was detected by confocal microscopy. Significant reduction in OHC loss rate with and without laser irradiation and also the potency in protecting the ribbon from Cis damage in all basal membrane turns was demonstrated. Consequently, the developed NIR‐based nanocomposite exhibited, resulting in improve therapeutic permeability into the inner ear and unique drug distribution across all inner OHCs turns.

## 5 Experimental Section

### Materials

Ascorbic acid (AA), Ammonium nitrate (NH4NO3), Tetraethyl orthosilicate (TEOS), Absolute ethanol, and Anhydrous cyclohexane were purchased from Sigma–Aldrich; Silver nitrate (AgNO3), Sodium borohydride (NaBH_4_), DEX, Saponin, were acquired from Fluka; Sodium hydroxide (NaOH), 3‐Aminopropyltriethoxysilane (APTES), and Cetyltrimethylammonium bromide (CTAB) were supplied from Argos, Gold (III) Chloride Trihydrate (HAuCl_4_.3H_2_O) were obtained from Macklin Inc.

### Methods—Synthesis of AuNRs

Seed‐mediated growth technique was used to synthesis the AuNRs in accordance with a previously published process with minor modifications.^[^ ^33^ ^]^ The detailed methods are provided in the Supporting Information.

Furthermore, m

### Synthesis of AuNR@MS

The Stöber method was used to cover the AuNR core with a layer of MS layer to create the core‐shell (AuNR@MS) particles. The synthesis is based on the hydrolysis of TEOS under alkaline circumstances using CTAB as a soft template and AuNR as a seed.^[^ ^19a^ ^]^ The detailed methods are provided in the Supporting Information.

### Synthesis of AuNR@MS‐NH_2_

Primary NH2 groups was added to the silica layer using 3‐aminopropyltriethoxysilane to modify the surface of the produced AuNR@MS. (100 mg) of synthesized AuNR@MS were mixed with (400 µL) of 3‐aminopropyltriethoxysilane in 20 mL of (100%) ethanol and incubated at 30 °C in water bath with stirring for 24 h. AuNR@MS‐NH_2_ was collected by centrifugation at 16 000 rpm, 20 min, at 18 °C and washed twice with (100%) ethanol.

### DEX Loading in AuNR@MS

The solvent immersion approach was utilized to load DEX one into AuNR@MS. (2 mg) of DEX (2 mg/mL) was dissolved in anhydrous cyclohexane solvent using sonication, followed by 20 min of sonication to suspend (1 mg) of AuNR@MS in the DEX solution.^[^ ^34^ ^]^ The suspension was then placed in a revolving wheel at 60 rpm, room temperature, for 24 h. The AuNR@DEX‐MS was extracted by centrifugation at 13 500 rpm for 10 min at room temperature, and then wash for two times. To quantify the drug loading content, DEX was eluted in ethanol at a concentration of (0.1 mg mL^−1^) while being repeatedly sonicated and vortexed for 1 h. later on, the nanoparticles were centrifuged, and the supernatant was measured at 242 nm using a Thermo Fisher Scientific 2000c UV–vis spectrophotometer. The loading content (LC%) and loading efficiency (LE%) of DEX were determined according to Equations: (3), (4).

(3) $$\begin{array}{cc} \mathrm{LE}\% & =\mathrm{total}\;\mathrm{quantity}\;\mathrm{of}\;\mathrm{DEX}\;\mathrm{input}\\ & \;-\mathrm{free}\;\mathrm{DEX}\;\mathrm{in}\;\mathrm{supernatant}/\mathrm{total}\;\mathrm{quantity}\;\mathrm{of}\;\mathrm{DEX}\;\mathrm{input}\times 100 \end{array}$$

(4) LC%=entrappedDEX/nanoparticleweight×100

### Synthesis (NPs/DEX)

The saponin was physically adsorbed to AuNR@DEX‐MS surface. (1 mg) of AuNR@DEX‐MS was dispersed in saponin DI water solution (2mg mL^−1^) for a few minutes using a sonication bath, and then it was stirred at room temperature for 24 h. The obtained NPs/DEX was centrifuged at 18 °C, 16 000 rpm for 20 min, washed twice to remove unreacted saponin, and then freeze‐dried.

### Photothermal Measurement of (NPs/DEX)

The photothermal effectiveness of NPs/DEX was assessed by three experiments: First, photothermal measurements were performed by irradiating (1 mL) of an aqueous dispersion of NPs/DEX with varying concentrations (0.25, 0.5, and 1 mg mL^−1^) with an NIR laser (980 nm, 1 W cm^−2^) for 300 s to study the concentration effect. In every 30 s, a thermal imaging picture was captured by a thermal imaging camera. Second, (1 mL) of an aqueous dispersion of NPs/DEX with the same concentration (0.5 mg mL^−1^) is irradiated with an NIR laser at various power densities (0.5, 1.0, and 1.5 W cm^−2^, 300 s) to study the laser power effect. Thirdly, (1 mL) of a (0.5 mg mL^−1^) suspension of NPs/DEX is irradiated with NIR laser at (980 nm, 1 W cm^−2^) to attain the plateau temperature, and then cooled down to room temperature naturally, to study the heating‐cooling cycle. Thermal images and temperature changes were recorded simultaneously every 30 s in all three studies using a digital thermometer camera.

### In Vitro Triggered Drug Release Via NIR

To imitate physiological pH, an in vitro drug release experiment was conducted using phosphate buffered saline (PBS) medium at pH (7.4) and body temperature (37 °C). At each time point, (1 mL) of dispersed NPs/DEX in PBS (1 mg mL^−1^) was agitated at 37 °C and pH (7.4) in a shaker bath. The drug release is quantified at 242 nm with a UV–vis spectrometer. Experiments on NIR laser‐controlled drug release utilized the same method. (1 mL) of dispersed NPs/DEX in pH 7.4 PBS (1 mg mL^−1^) was irradiated with an NIR laser at (980 nm, 1 W cm^−2^, 5 min) at a predetermined time interval.

### Characterization

(5 µL) of nanoparticles was dispersed in ethanol or an aqueous solution on carbon‐coated copper grids (200 mesh; Ted Pella, Inc., U.S.A.), dried in air, and the shape, size, and aspect ratio of nanoparticles were analyzed using the JEM‐1400 Plus TEM. The hydrodynamic size, and PDI of the nanoparticles were analyzed with Malvern Zeta Sizer ZS (PCS, Malvern Instruments Ltd). In addition, FTIR spectra were scanned from 4000 to 400 cm^−1^ with a PerkinElmer Spectrum Two in order to examine the surface structure of drugs, polymers, and manufactured nanocomposites with diverse surface modifications.

### In Vitro Cytotoxicity and Hearing Protection of Synthesized NPs/DEX Nanocomposite

HEI‐OC1 cells were cultured in antibiotic‐free Dulbecco's modified Eagle's medium with (10%) fetal bovine serum (FBS; Gibco) and high glucose (DMEM; Gibco). CCK8 (Beyotime) was utilized to assess the cytotoxicity of NPs/DEX on HEI‐OC1 cells. HEI‐OC1 cells were seeded in the 96‐well plates at a density of 5× 10^3^ cells/well and incubated for 24 h. The medium was then substituted with Cis, saponin, NPs/DEX, and DEX medium of different concentrations. The control consisted of a medium free of NPs or DEX drug molecules. (10µL) of CCK‐8 was added to the medium after 24h initial incubation, and an additional 1.5 h incubation was applied to the plates before measuring their absorbance at 450 nm using a microplate reader (BioTek, Synergy2).

CCK8 was also used for the cytoprotecting measurement of NPs/DEX in HEI‐OC1 cells against Cis. HEI‐OC1 cells were seeded as described previously and incubated for 24 h at (80%) confluence. DEX (100 ng mL^−1^) medium without FBS was regard as the optimal protective concentration against Cis. The media was subsequently replenished with culture medium, NPs/DEX medium (equivalent to 100, 200, and 300 ng mL^−1^ DEX), and (100 ng mL^−1^) DEX medium without FBS for 5 h. After the application of NIR laser with (980 nm, 0.3 W cm^−2^, 10 s), Cis at a specific concentration (3000 µm) was applied to each well to make the ultimate concentration as 30 µm. As a control, the medium without DEX or NPs was used. After 24 or 48 h of incubation, the viability of the cells was determined as described previously.

### Cisplatin‐Induced Hearing Loss Model

All animal experiments were in accordance with the ethical guidelines set by the Institutional Ethnics Committee of Eye, Ear, Nose and Throat Hospital of Fudan University for the care of laboratory animals. To establish the CIHL model, guinea pigs were hypodermic administered Cis. Guinea pigs weighing between 250 and 350 g were housed in a specially made insulated cages to keep warm. Briefly, (10 mg Kg^−1^) Cis was hypodermic administered to the back of guinea pigs to induce deafness. ABR examinations were applied to evaluate hearing function before and after Cis administration.

The animals were divided into the following groups: the control group, in which the animals was not exposed to Cis but only received a sham IT; the Cis group, in which the animals received only an IT of 100 µL normal saline solution; the Cis+DEX group and Cis+DEX+Laser group, in which the animals received an IT of 100 µL DEX (100µg mL^−1^); the Cis+NPs/DEX+DEX group and Cis+NPs/DEX+DEX+Laser group, in which each animal received an IT of 100 µL NPs/DEX+DEX (equivalent to NPs/50 µg mL^−1^ DEX+ 50 µg mL^−1^ DEX solution; total = 100 µg mL^−1^ DEX) in saline solution; the Cis + NPs/DEX group and Cis + NPs/DEX + Laser group, in which the animals received an IT of 100 µL per ear NPs/DEX (100 µg mL^−1^ DEX) in saline solution; the Cis + NPs + DEX group, in which the animals received an IT of 100 µL per ear NPs+DEX (equivalent NPs and 100 µg mL^−1^ DEX) in saline solution. All Cis group received the hypodermic administered of Cis (10 mg Kg−1). All animals were anaesthetized and bilaterally administered with the corresponding injection. The animals were then placed in a uniform position for 30 min. The laser group was pretreated with optimized laser power intensity (980 nm, 0.3 W cm^−2^ for 10 s) after receiving IT with NPs/DEX or DEX. Two days after IT, injections of (10 mg Kg^−1^) Cis were hypodermic administered to each of the animal groups. The placebo group received IT without Cis. ABR thresholds were measured at baseline and 4 days after Cis injection. Immunostaining was then used to characterize the morphology of hair cells and synaptic ribbons in the acquired cochleae.

### ABR Recordings

Guinea pigs were anaesthetized and cared for as previously reported.^[^ ^6b^ ^]^ The animals suffering from otitis media were not included. The detailed methods are provided in the supplementary materials. 

### Histological and Immunohistochemical Examinations

Cochleae were extracted from animals at specific times and the cochleae were fixed as previously described.^[^ ^6b^ ^]^ The detailed Immunohistochemical methods are provided in the Supporting Information. Laser scanning confocal microscopy (Leica, SP8) was used to capture the fluorescence pictures, which were then processed in ImageJ (Fiji). For OHC quantification, DAPI‐ and phalloidin‐labeled hair cells were counted per 250 µm for OHC quantification. The average number of OHCs in each turn was computed in each group. Synaptic ribbons (anti‐CtBP2, gray) were counted in three independent randomly selected areas per sample to produce an average value. This procedure was performed on fixed‐size sections that included specific six IHCs.

### Statistical Analysis

All statistical analyses were performed using GraphPad Prism (GraphPad Software). The data are shown in the form of means ± standard deviations. When comparing two independent samples, statistical analysis was performed using an unpaired *t*‐test, with or without Welch's correction, as appropriate. A one‐way analysis of variance (ANOVA) was used to determine the distinctions among the groups. *p* < 0.05 was considered to be statistically significant.

## Conflict of Interest

The authors declare no conflict of interest.

## Author Contributions

R.A.M., J.W., and M.X. contributed equally to this work and should be considered co‐first authors. W.W., Y.S., and H.Z. jointly supervised this work. R.A.M. synthesized and characterized all nanoparticle samples, data curation, formal analysis, writing – original draft, writing – review & editing. J.W. conducted in vitro and in vivo study, data curation, formal analysis, writing – review & editing. M.X. conducted in vitro and in vivo study, and data curation. J.M.R. supervision, writing – review & editing. W.W. supervision, writing – review & editing. Y.S. supervision, writing – review & editing, funding acqusition. H.Z. supervision, project administration, writing – review & editing, funding acquisition.

## Supporting information



Supporting Information

## Data Availability

The data that support the findings of this study are available from the corresponding author upon reasonable request.
