# Supplementary material for: Programmable NIR Responsive Nanocomposite Enables Noninvasive Intratympanic Delivery of Dexamethasone to Reverse Cisplatin Induced Hearing Loss
Source: Adv Sci (Weinh). 2024 Nov 21;12(24):2407067. doi: 10.1002/advs.202407067 (PMC12199587; doi:10.1002/advs.202407067)
Supplement: Supplementary file 1 — Supporting Information [file ADVS-12-2407067-s001.docx]

Supporting Information

**Programmable NIR Responsive Nanocomposite Enables Noninvasive Intratympanic Delivery of Dexamethasone to Reverse Cisplatin Induced Hearing Loss**

*Rawand A. Mustafa ^1, 4, 6ᵻ^, Jiali Wang ^1, 2, 3, 5ᵻ^ , Mengzhao Xun ^1, 2, 3 ,5ᵻ^, Jessica M. Rosenholm ^4^, Wuqing Wang ^1, 2^*, Yilai Shu ^1, 2, 3, 5^*, Hongbo Zhang ^4,6^**

**^1^** ENT institute and Department of Otorhinolaryngology, Eye & ENT Hospital, Fudan University, Shanghai 200031, P. R. China

**^2^** NHC Key Laboratory of Hearing Medicine Research, Shanghai 200031, P. R. China

**^3^** State Key Laboratory of Medical Neurobiology and MOE Frontiers Center for Brain Science, Fudan University, Shanghai 200031, P. R. China

**^4^** Pharmaceutical Sciences Laboratory, Faculty of Science and Engineering, Åbo Akademi University, Turku, 20520, Finland

**^5^** Institutes of Biomedical Sciences, Fudan University, 200032, P. R. China

**^6^** Turku Bioscience Centre, University of Turku and Åbo Akademi University, Turku, 20520, Finland


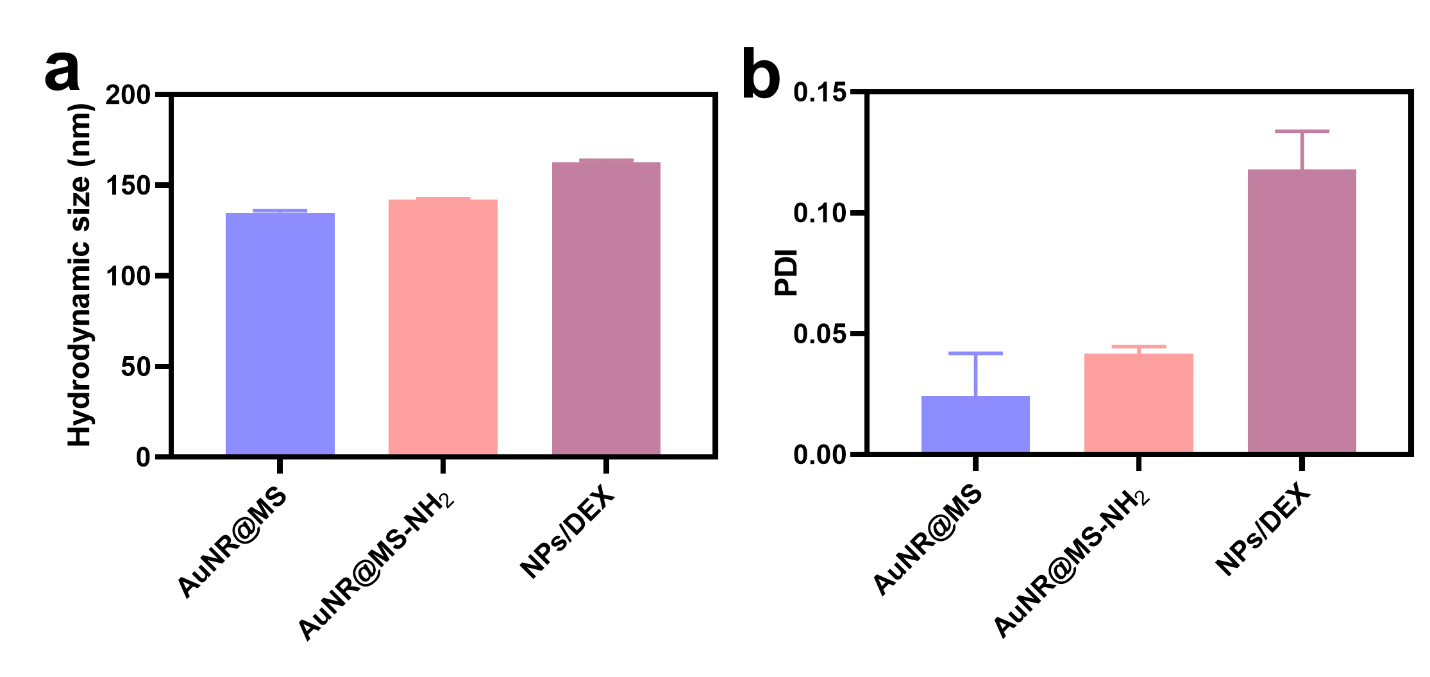


**Figure S1.** DLS measurements of the hydrodynamic size and polydispersity index (PDI) of AuNR@MS, AuMR-MS-NH_2_, and NPs/DEX. a) Hydrodynamic particle size of AuNR@MS, AuNR@MS-NH_2_, and NPs/DEX. b) PDI of AuNR@MS, AuNR@MS-NH_2_, as well as NPs/DEX.


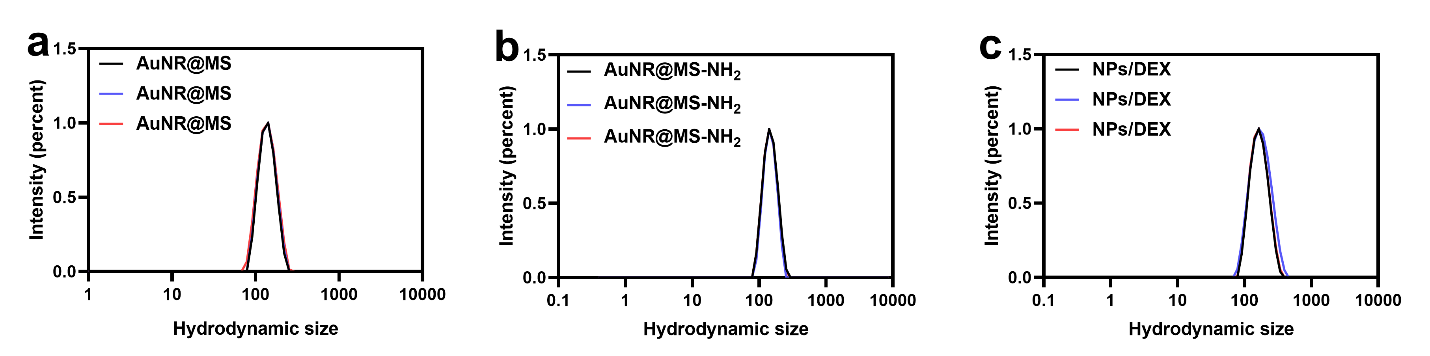


**Figure S2.** Measuring the hydrodynamic size distribution (intensity) of AuNR@MS, AuNR@MS-NH_2_, and NPs/DEX with DLS. a) Measurements of particle size distribution of AuNR@MS. b) Measurements of particle size distribution of AuNR@MS-NH_2._ c) Measurements of particle size distribution of NPs/DEX.


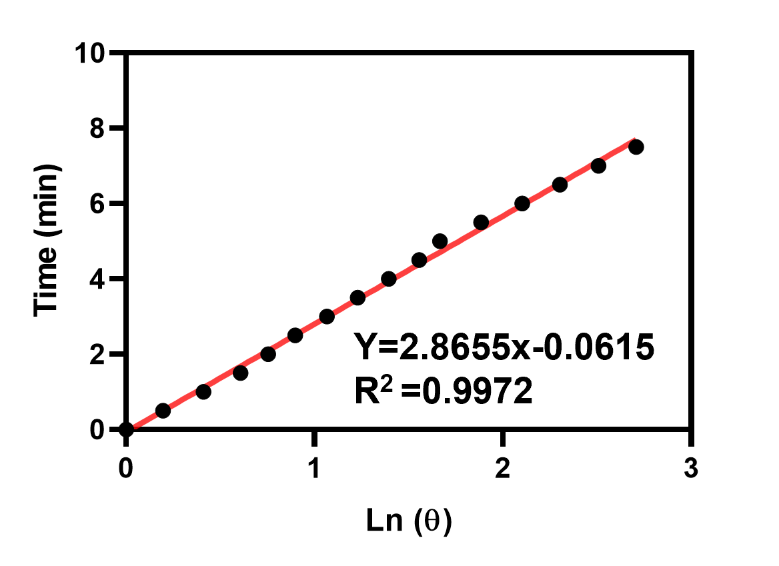


**Figure S3.** Data for linear time versus -lnθ derived from the cooling period of Figure 2d.


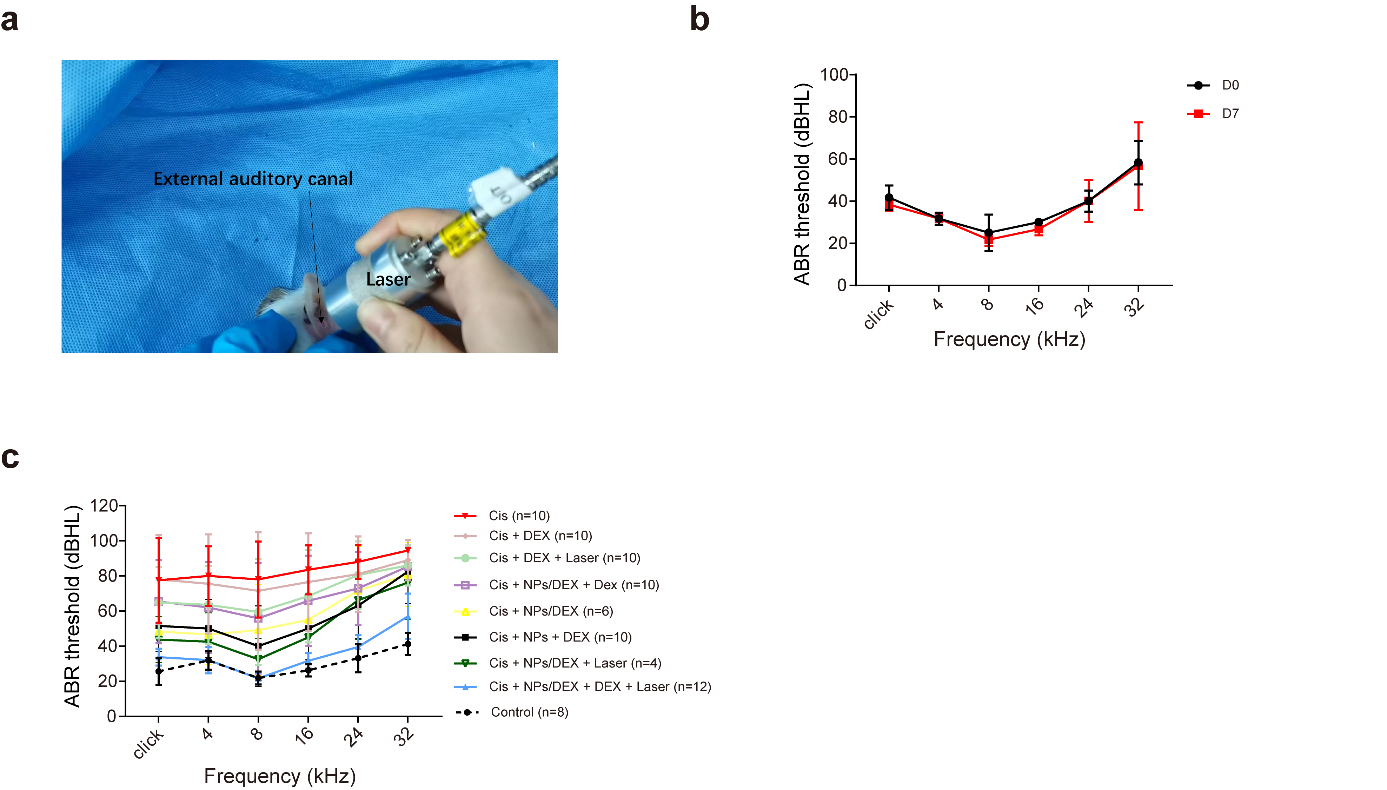


**Figure S4.** a) Sites of laser irradiation in animal experiment. b) The ABR threshold of guinea pigs before and after intratympanic injection NPs/DEX with Laser on day 0 and day 7 (n = 3). c) ABR threshold of guinea pigs on day 4 following Cis overexposure at various frequencies in the different groups.

**Experimental Section**

*Methods—Synthesis of AuNRs*

Initially, the seed solution was prepared by adding (25µL) of (50 mm) HAuCl_4_.3H_2_O to (4.7 mL) of (0.1m) CTAB in a 10mL glass tube, followed by gentle stirring in a 30 °C water bath for 2–5 min or more till a clear solution was obtained. Subsequently (300µL) of (10 mm) of ice‐cold NaBH_4_ was added with vigorous stirring for 1–2 min at 30 °C in a water bath, all at once. The seed solution was stored at 30 °C in a water bath without shaking. The color changed quickly to light brown.

Furthermore, by adding (1000 µL) of (50 mm) HAuCl_4_.3H_2_O and (1900 µL) of (1 m) HCL to (100 mL) of (0.1 m) CTAB in a glass bottle, followed by 5–10 min of gentle stirring in a 30 °C water bath, a growth solution was prepared. After this, (1200 µL) of a (10 mm) AgNO_3_ solution was added, and the mixture was stirred for a few seconds. The solution became colorless upon the addition of (1000 µL) of (100 mm) ascorbic acid (AA) and a few seconds of gentle stirring. Lastly, (240 µL) of the prepared seed solution was added to the growth solution, gently mixed for 10–20 s to ensure proper mixing, and then incubated at 30 °C for 24 h without shaking. Gradually, the color of the mixture changed to a light brown. To eliminate unreacted CTAB and other impurities, the produced AuNR was centrifuged once at 16 000 rpm for 20 min at 18 °C, then washed once with deionized water and centrifuged at the same settings. The AuNR was then re‐dispersed in (10 mL) of deionized water and kept at +4 °C for future use.

*Synthesis of AuNR@MS*

Initially, (10 mL)of the as‐prepared AuNR suspension was adjusted to a pH of 10–11 using (0.1 m) NaOH. To equilibrate the suspension temperature, the reaction is then transferred to a round‐bottom flask and stirred at 300 rpm in a 30 °C water bath for 20 min. Then (120 µL) of freshly made (20% TEOS) in absolute ethanol solution was added step‐by‐step (within 10–15 min) while stirring and continued for 24 h. Following 24 h, the AuNR@MS was centrifuged for 20 min at 16 000 rpm and 18 °C. To remove excess CTAB and unreacted silica, the mixture was then re‐dispersed in (30 mL) of a (1% NH4NO3) ethanol solution and stirred vigorously for 6 h twice in a 30 °C water bath. The produced AuNR@MS was subsequently rinsed with (100%) ethanol and centrifuged at the same speed to eliminate excess NH4NO3. AuNR@MS was then re‐dispersed in absolute ethanol and stored at +4 °C for future use.

*ABR Recordings*

ABR was recorded through an opened field speaker by the RZ6 signal processor at various frequencies, including clicks and tones (4, 8, 16, 24, and 32 kHz), at intensities decreasing from 90 to 20 dB in 5 dB steps. The thresholds were recorded using BioSig software (TDT, Alachua, FL). When no wave was detectable at 90 dB SPL, the ABR thresholds were recorded as 95 dB SPL.

*Immunohistochemical Examinations*

The basal membranes were sliced and immersed in PBS (1% Triton X‐100, 10% BSA) at 37 °C for 1 h. Hair cell damage was measured for each group. Nuclei were labeled with 4′,6‐diamidino‐2‐phenylindole (DAPI; Sigma‐Aldrich, D9542), and phalloidin (1:1000) was used to stain the cilia of cochlear hair cells. IHCs and synaptic filaments were identified using Myosin VIIA (1:500; 25–6790, Proteus Biosciences) and CtBP2 (1:200, BD Biosciences). The observation was conducted using secondary antibodies with fluorescence.
